# The GCTx format and cmap{Py, R, M, J} packages: resources for optimized storage and integrated traversal of annotated dense matrices

**DOI:** 10.1093/bioinformatics/bty784

**Published:** 2018-09-10

**Authors:** Oana M Enache, David L Lahr, Ted E Natoli, Lev Litichevskiy, David Wadden, Corey Flynn, Joshua Gould, Jacob K Asiedu, Rajiv Narayan, Aravind Subramanian

**Affiliations:** 1 The Broad Institute, Cambridge, MA, USA; 2 Department of Computer Science and Engineering, University of Washington, Seattle, WA, USA; 3 MediaSilo, Boston, MA, USA

## Abstract

**Motivation:**

Facilitated by technological improvements, pharmacologic and genetic perturbational datasets have grown in recent years to include millions of experiments. Sharing and publicly distributing these diverse data creates many opportunities for discovery, but in recent years the unprecedented size of data generated and its complex associated metadata have also created data storage and integration challenges.

**Results:**

We present the GCTx file format and a suite of open-source packages for the efficient storage, serialization and analysis of dense two-dimensional matrices. We have extensively used the format in the Connectivity Map to assemble and share massive datasets currently comprising 1.3 million experiments, and we anticipate that the format’s generalizability, paired with code libraries that we provide, will lower barriers for integrated cross-assay analysis and algorithm development.

**Availability and implementation:**

Software packages (available in Python, R, Matlab and Java) are freely available at https://github.com/cmap. Additional instructions, tutorials and datasets are available at clue.io/code.

**Supplementary information:**

Supplementary data are available at *Bioinformatics* online.

## 1 Introduction

Computational analysis of datasets generated by treating diverse cell types with pharmacological and genetic perturbagens has proven useful for functional relationship discovery (Hughes *et al.*, 2000; Lamb *et al.*, 2006; Weinstein *et al.*, 1997). To enable such discovery, the NIH Common Fund’s Library of Network-Based Cellular Signatures (LINCS) has brought together several high-dimensional assays to systematically characterize the effects of perturbagens on human cells (Keenan, 2018).

Additionally, the scale of the perturbation-based datasets produced has multiplied in recent years to encompass millions of samples. Such large-scale, holistic representations of perturbation provide an incredible opportunity for systems-based research on health and disease, but also introduce data management challenges unique to perturbation-based functional compendia. In particular, two challenges that are crucial to address in order to facilitate analysis of these initially heterogeneous data are standardized formatting of assay output and ease of access to arbitrary ranges of output datasets.

While the more mature field of DNA sequencing has largely converged on a standard set of file formats and data types, raw forms of perturbational data are more diverse and can range from flow cytometry readouts for mRNA (Subramanian *et al.*, 2017) to mass spectrometry traces for protein phosphorylation (Abelin *et al.*, 2016) to quantitative data extracted from microscopy images for morphological profiling (Bray *et al*, 2016). Each of these diverse data types has associated metadata, and so additional relevant metadata annotations on literature pathways (Liberzon *et al.*, 2011), drug targets and mechanisms of action (Corsello *et al.*, 2017), are also key to interpretation of analysis results.

Although the LINCS consortium thoughtfully considered the challenges in integrating heterogeneous data in its establishment of standards for metadata prior to public data deposition (Vempati et al., 2014), adoption of a standard for data deposition in itself does not necessarily ease access for computation during exploratory data analysis. To address this, we present the GCTx file format along with open-source software packages that we have developed. GCTx relies on robust HDF5 technology to make large, dense matrices of data and metadata annotations easy to store and explore. Importantly, the format’s utility is not just theoretical: to date, we have aggregated, analyzed, and publicly distributed millions of profiles‘ worth of data from LINCS and other large compendia (Supplementary Material S1).

## 2 The GCTx format and code libraries

**Fig. 1.**
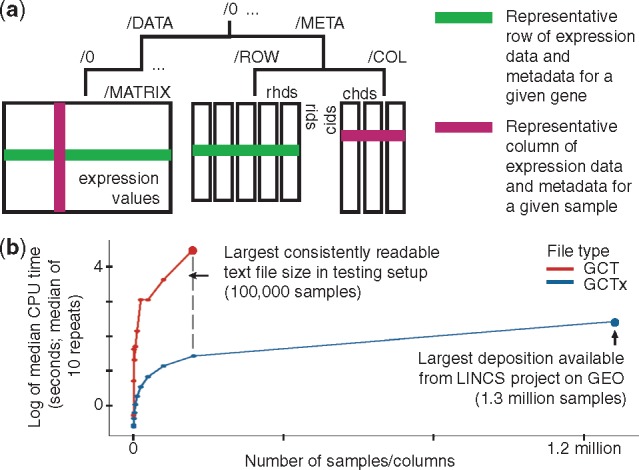
(**a**) Schematic of a GCTx file. (**b**) Parse times are faster for GCTx files compared with text-based files; more details in Supplementary Material S3

***Choice of a data representation technology.*** Text-based formats like GCT (Supplementary Material S2) have long been used in gene expression analysis (Eisen *et al.*, 1998). However, the dramatically increasing size of datasets in more recent years has made storage as plain text impractical (Fig. 1B). In addition, without a governing data model, text formats cannot efficiently represent relationships between rows, columns and metadata. Furthermore, text formats make it cumbersome to retrace the provenance of an element in the data matrix as it passes through multiple stages of a data processing pipeline, which is an important requirement for reproducibility.

***The GCTx format***. The format we developed that addresses these issues is a schema built on HDF5 that we term GCTx. HDF5 supports a platform-independent file format capable of unlimited size, rapid read/write capabilities and selective parsing of a subset of a dataset without loading the entire file into memory first (The HDF Group, 1997–2018). In addition, HDF5 has a vibrant developer community that supports multiple programming languages and operating systems. GCTx adopts a lightweight, shallow hierarchy optimized for representing matrices and associated annotations while retaining several of the key benefits of HDF5’s infrastructure. This shallow hierarchy of component nodes decreases random access time when compared to deeply nested alternatives and also enables simple and efficient extension in the data matrix (appending it to the /MATRIX node) or dataset (incrementing the numerically indexed group name to ‘/1’, ‘/2’, etc. and then appending data and metadata) dimensions (Fig. 1A). More broadly, the standardization of data and metadata representation that GCTx provides frees developers from having to repeatedly customize their analytical code and to think about how to optimally use or represent data in HDF5. This is important because the HDF5 format only provides a generic data model; standardizing the representation of data and metadata consequently encourages reproducibility of data analyses.

***Open source software packages integrated with GCTx***. To facilitate adoption of the GCTx format with existing bioinformatics and data science tools, we also developed four open source software packages in Python (‘cmapPy’), R (‘cmapR’), Matlab (‘cmapM’) and Java (‘cmapJ’), which simplify input, output, conversion and analysis of GCTx files by representing these file inputs as native data structures readily compatible with powerful data analysis tools.

## 3 Conclusions

We present GCTx, an HDF5-based file format designed for efficient storage and rapid access of dense data matrices paired with metadata annotations. The format’s ability to store multiple distinct datasets and annotations enable a single file to contain an entire workflow’s worth of content, which aids reproducibility in analyses and collaboration. Importantly, the format's utility is not just theoretical: to date, we have compiled ∼1.3 million samples and made them freely available in the GCTx format (Supplementary Material S3).

Worth noting is that HDF5-based formats have previously been used in genomics (Millard *et al.*, 2011; Sommer *et al.*, 2013); however, these prior formats differ from GCTx in that most of them involve using deep hierarchies to store a variety of experimental design and modeling data with assay output. While this can be a useful structure, our primary needs deviated sufficiently from the features of other HDF5-based formats to merit the development of our own format. Additionally, although relational databases and cloud-based object stores are also capable of storing and efficiently serving massive datasets, we have found that--even as cloud-based object stores become more commonplace--users still request downloadable file-based representations of data for use on their personal computers or traditional login servers. Although this may change over time, we consequently decided that a file-based format would best address the majority of current user needs. To ease adoption of GCTx, we also present four open-source packages that make GCTx straightforward to incorporate with existing tools.

## Supplementary Material

Supplementary Data
