# Supplementary material for: The GCTx format and cmap{Py, R, M, J} packages: resources for optimized storage and integrated traversal of annotated dense matrices
Source: Bioinformatics. 2018 Sep 10;35(8):1427–9. doi: 10.1093/bioinformatics/bty784 (PMC6477971; doi:10.1093/bioinformatics/bty784)
Supplement: Supplementary Data [file bty784_supplementary_data.docx]

**Supplement 1. Publicly available datasets using GCTx**

## GCTx’s capability to portably represent diverse data types has lead to its adoption by many projects; currently, publicly available data in GCTx format include L1000 gene expression datasets from the Connectivity Map (GSE70138; GSE92742), phosphorylation and chromatin datasets from LINCS (GSE101406; Litichevskiy *et al.*, 2017), and RNA-Seq data collated and processed by the ARCHS4 project (Lachman *et al.,* 2017). Descriptions of these data and their interoperability with the GCTx code libraries are provided at [clue.io/code](https://clue.io/code).

**Supplement 2. Format of a GCT file**

GCT is a tab-delimited text file containing information in the following format:

**
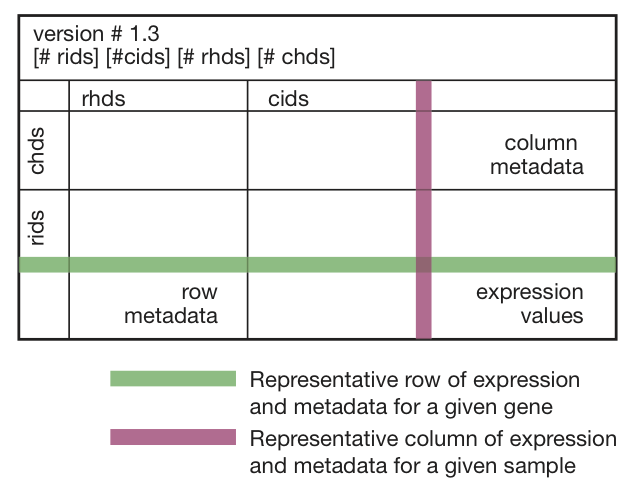
**

The GCTx format (Figure 1a) contains the same content but with improved performance benefits.

**Supplement 3. GCTx performance benchmarking**

To assess the performance of GCTx files compared to their text file equivalents, we performed benchmarking tests that parsed various sizes of text (GCT) and GCTx files of identical content (Figure 1B in main text). Benchmarking was performed on an Amazon Web Services EC2 instance with a locally mounted solid state drive (instancetype = i3.16xlarge, memory = 488 GB, vCPU = 64). For each of the software packages available (cmapM, cmapPy, cmapR), the same set of representative files of expression data was parsed 10 times (cache cleared between sequential reads). The median times for all parsing operations performed in Python are plotted in Figure 1B in main text; similar trends were observed for the Matlab and R implementations. The text-based GCT files become considerably slower to read from and write to as file sizes increase beyond tens of thousands of columns/samples, and were not performant on the testing setup used as file sizes increased beyond a hundred thousand samples/columns. However, GCTx can parse and write equivalent and much larger content considerably faster, and continues to be perform efficiently for much larger file sizes. For example, medium CPU time to parse the largest LINCS file published to GEO (accession GSE 92742) with methods from cmapPy took 252.2 seconds on testing setup.

**Additional References**

Lachmann,A. *et al.* (2017) Massive Mining of Publicly Available RNA-seq Data from Human and Mouse. *bioRxiv*, 189092.

Litichevskiy, L., Peckner, R., *et al*. (2017) “A Library of Phosphoproteomic and Chromatin Signatures for Characterizing Cellular Responses to Drug Perturbations.” *bioRxiv*.
